# Atrial reconstruction, distal gastrectomy with *Ante-situm* liver resection and autotransplantation for hepatocellular carcinoma with atrial tumor thrombus: A case report

**DOI:** 10.1097/MD.0000000000025780

**Published:** 2021-05-14

**Authors:** Tuerhongjiang Tuxun, Shadike Apaer, Gang Yao, Zhipeng Wang, Shensen Gu, Qi Zeng, Aidan Aizezijiang, Jing Wu, Nuerzhatijiang Anweier, Jinming Zhao, Tao Li

**Affiliations:** a Department of Liver Transplantation & Laparoscopic Surgery, Center of Organ Transplantation, The 1st Affiliated Hospital of Xinjiang Medical University; b Clinical Research Center of Hydatid and Hepatobiliary Disease, The 1st Affiliated Hospital of Xinjiang Medical University; c School of Language, Shanghai University of International Business and Economics, China.

**Keywords:** *ante-situm*, autotransplantation, hepatocellular carcinoma, radical resection, tumor thrombi

## Abstract

**Rationale:**

Hepatocellular with tumor thrombi extending into 3 hepatic veins (HVs) and right atrium presents as a real clinical challenge. We report the first documented case of surgical resection of an advanced hepatocellular carcinoma (HCC) with extensive invasion to distal stomach, atrium and hepatic vasculatures.

**Patient concerns:**

We present a case of 48-years old man with abdominal mass accompanying shortness of breath after activities.

**Diagnoses:**

Preoperative examination revealed giant HCC with tumor thrombi extending into portal vein, HVs, inferior vena cava, and atrium.

**Interventions:**

Distal stomach involvement was confirmed at surgery and, distal gastrectomy, atrial reconstruction and *ante-situm* liver resection and autotransplantation under cardio-pulmonary bypass were performed.

**Outcomes:**

The operation time was 490 minutes, extracorporeal circulation time 124 minutes, and anhepatic time 40 minutes. Postoperative follow-up revealed normal hepatic and cardiac function with no sign of recurrence.

**Lessons:**

This case illustrates that the extensive invasion of HCC to major vasculature and adjacent organs may not necessarily preclude the liver autotransplantation with multi-visceral resection as the treatment option of extremely advanced HCC patients.

## 1 Introduction

Hepatocellular carcinoma (HCC) with macrovascular tumor thrombus (MTT) presents as an advanced stage and is associated with poor prognosis.^[1]^ Portal vein tumor thrombosis (PVTT) is the most commonly seen, however, tumor thrombosis in right atrium and/or inferior vena cava (IVC) are rarely reported with the incidence of 2%.^[2]^ Despite high morbidity and mortality in patients with MTT, surgery continues to be the radical modality and provides higher survival rates compare to nonsurgical treatment.^[3]^ The presence of MTT is contraindication to liver transplantation and therefore radical liver resection with thrombectomy is the only surgical option.^[4]^ However, only a small number of patient could benefit from surgery due to severe liver cirrhosis and/or extensive invasion to hilum and hepato-caval regions.^[5]^*Ante-situm* liver resection and autotransplantation was introduced to increase the resectibility of some otherwise irresectable tumors with conventional resection techniques.^[6]^ Herein, we present first documented case of *ante-situm* liver resection and autotransplantation with *cavo-atrial* thrombectomy and distal gastrectomy in an advanced HCC patient. The HCC lesion severely infiltrated to stomach with tumor thrombus (TT) severely extending into 3 hepatic veins (HVs), IVC, right atrium and portal vein.

## 2 Case report

**Figure 1:**
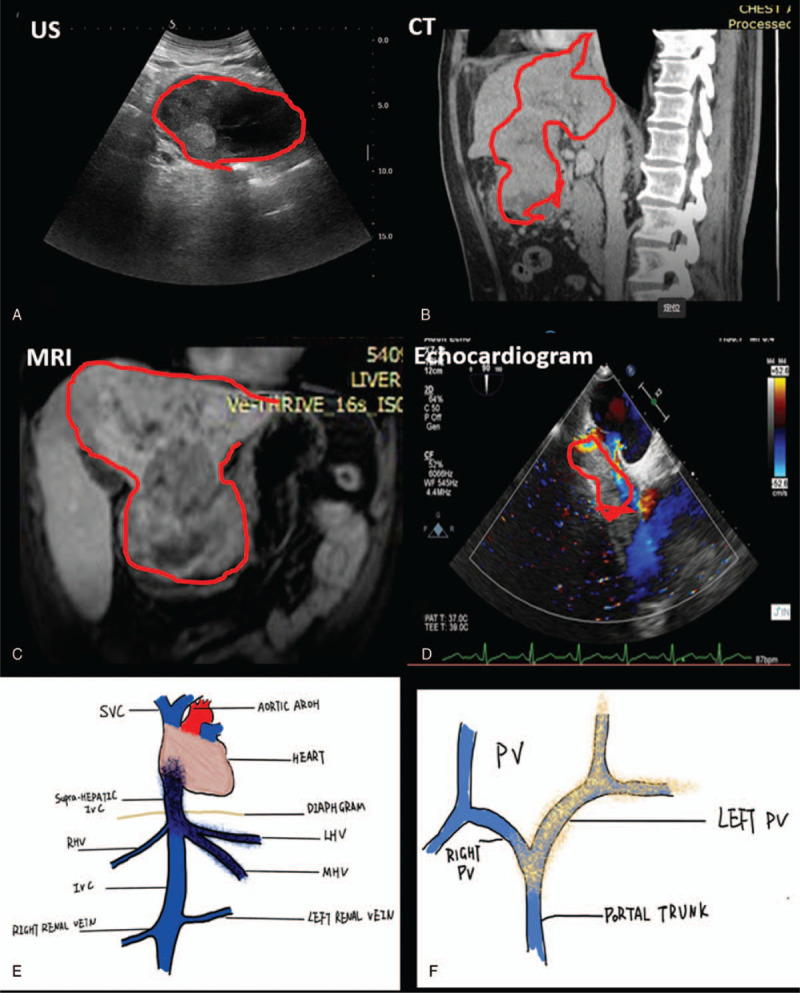
Preoperative assessment of the HCC lesion. A, ultrasonography imaging; B, CT scan showed a giant HCC lesion with tumor thrombi extending into right atrium; C, MRI scan showed huge mass in left lobe; D, echocardiogram showed a 5.0 cm × 2.8 cm mobile mass in supra-hepatic IVC and right atrium; E, the extension of tumor thrombi in IVC, hepatic veins and right atrium; F, the extension of tumor thrombi into portal vein.

A 48-years old HCC patient with chronic hepatitis B virus infection (having lasted for nearly 20 years) and liver cirrhosis was transferred to our department for possible surgical resection. Physical examination found an immobile abdominal mass at left upper quartile with no tenderness. Laboratory results showed normal liver function except with slightly increased level of aspartate aminotransferase (72 U/L). Tumor marker analysis showed increased levels of CA125 (164 U/ml) and serum ferritin (456 ng/ml) but normal α-fetoprotein. COVID-19 antibody and throat swab nucleic acid test were all negative. Ultrasound showed a giant hepatic mass protruding to abdominal cavity with HVs and portal veins tumor thrombosis (Fig. 1A). Computed tomography and magnetic resonance imaging scan showed a giant HCC lesion 20 cm × 11 cm with tumor thrombosis extended into IVC, left hepatic vein (LHV), middle hepatic vein (MHV), orifice of right hepatic vein (RHV), left portal vein (LPV), and portal trunk (Fig. 1B-C). Preoperative echocardiogram confirmed a 5.0 cm × 2.8 cm mobile mass in supra-hepatic IVC and right atrium (Fig. 1D). Gastroscope showed moderate esophageal gastric varicose with no intraluminal occupational lesion. Ultrasonic elastic imaging showed F3 stage liver fibrosis. Pulmonary computed tomography scan and function test showed no abnormality. The HCC lesion with tumor thrombi extension was schematized (Figure 1E-F). Preoperative multi-disciplinary team including hepatic surgeon, cardiovascular surgeon, anesthesiologist, radiologist, pathologist, and hepatologist carefully reviewed all preoperative results and made the decision for *ante-situm* resection and thrombectomy. Taking the critical involvement of HCC lesion to hepato-caval region type 3 atrial tumor thrombosis, distal stomach and cirrhotic background, *en bloc* resection including *ante-situm* left hepatectomy with reimplantation of right liver, cava-atrial thrombectomy, and distal gastrectomy under cardio-pulmonary bypass was planned.

**Figure 2:**
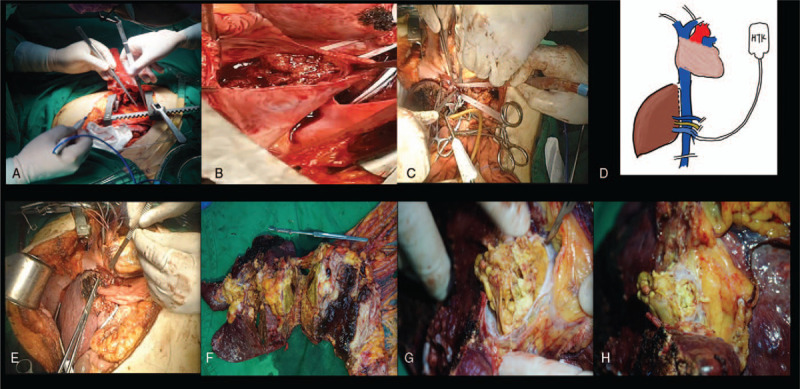
*Ante-situm* resection of HCC with tumor thrombi in IVC, right atrium and portal vein. A, sternotomy was performed in order to develop cardiopulmonary bypass; B, the tumor thrombi in right atrium was exposed after opening the atrium; C, HTK perfusion via portal vein; D, schematic *ante-situm* resection of the liver; E, reimplanted remnant liver; F, resected specimen with tumor involved into stomach; G, tumor thrombi in right atrium; H, tumor thrombi in portal vein.

A right subcostal inverse “L” shaped incision was taken as first step. At laparotomy, a giant tumor at the left lobe of cirrhotic liver with very little ascites was found. The lesion protruded and severely infiltrated and adhered to distal stomach. No extra-peritoneal metastasis was found. For the purpose of *en bloc* resection, distal gastrectomy and the gastro-enteric Roux-en-Y anastomosis was performed. Then, the hilar region was dissected and hepatic artery portal vein, common bile duct, and infra-hepatic IVC were identified and suspended. The left hepatic artery and aberrant left hepatic artery was ligated. Hepatic parenchymal was performed by using crush clamping technique in combination with ultrasonic scalpel. When hepato-caval region was exposed, the dissection was discontinued. Following laparotomy, a median sternotomy was taken to prepare for prompt CPB. The superior vena cava (SVC), ascending aorta and right femoral vein were cannulated to ready to start the CPB. The portal trunk was clamped blow the portal thrombus, meanwhile, the right portal vein was transected and clamped in order to completely remove the thrombus. Thereafter, CPB was started and atrium opened and RHV was disconnected to IVC. An ice bag was placed at the right upper abdomen region and 2000 ml chilly HTK solution was perfused via right portal vein. Then the rest of the liver parenchyma was dissected and the tumor thrombosis in IVC, right atrium, MHV, LHV, and portal vein were completely removed along with the distal stomach. The atrium and IVC were reconstructed and followed by the end-to side anastomosis of MHV to IVC. Portal trunk was anastomosed to right portal vein with end-end fashion (Fig. 2A-E). The operation time was 490 minutes, CPB time was 124 minutes and an-hepatic time was 45 minutes.

The macroscopic findings of resected tumor measured 15.5 ^∗^ 12.5 ^∗^ 6.5 cm^3^ and the TT in atrium measured 5.0 ^∗^ 3.0 cm^2^. Post-operative histological diagnosis showed moderately differentiated HCC had invaded the serosa of stomach with hepatic tumor thrombosis in IVC, LHV, MHV, and portal vein were found hepatic cirrhosis and intravascular TT (Fig. 2F-H). No positive resection margin or local lymph node metastasis were found microscopically.

Postoperative recovery was uneventful. The patient was discharged after 12 days and was good condition and disease free 2 months after his surgery.

Our institutional review board was waived due to the retrospective nature of the study. Written informed consent was obtained from the patient for the publication of this case report.

## 3 Discussion

Advanced HCC with tumor thrombi in IVC and/or right atrium is clinically rare and associated with poor prognosis.^[7]^ Management is challenging and treatment modality should be chosen individually based upon patients’ general conditions, hepatic function, size, and site of thrombi and as well as metastatic status.^[8]^ Since HCC with MTT is contraindicating to liver transplantation, radical resection is only the curative option whenever the surgery is considered.^[9]^*Ante-situm* liver resection and autotransplantation was firstly introduced by Sauvanet et.al^[6]^ and practiced for surgical resection of hepatic tumors that are otherwise unresectable with conventional hepatic surgery. In this report, the HCC lesion is huge with involvement to right atrium, hepato-caval region, and first hilum. Wide access on all parts of the liver is provided by section of supra-hepatic *vena cava* and opening of atrium, but the continuity of the portal triad is preserved. Despite this technique is more aggressive than conventional hepatectomy, but the reported morbidity and mortality was acceptable in high volume centers.^[10]^ Our previous experience with the practice of ex vivo liver resection and autotransplantation with large numbers of advanced hepatic lesions has smoothed both surgical practice and postoperative management of current case.^[11,12]^

The management of HCC with TT extending into IVC and / or RA are clinically challenging, the option for appropriate treatment is a dilemma. Despite of reported usefulness of TACE,^[13]^ RFA,^[14]^ and radiotherapy^[15]^ alone in such cases, surgery seems to demonstrate its effectiveness with improved survival rate. As far as surgery concerned, timing and long-term outcome are the critical question should be answered. In this report, the TT in right atrium is mobile and pulmonary embolism may occur if treated untimely. With support to our report, study showed that HCC patients with atrial thrombus usually die with in short period of time because of pulmonary embolism, heart failure or cancer progression.^[16]^ Preoperative assessment of “size” and “quality” for remaining liver is vital importance in such cases. Liver function of this patient was categorized into Child-Pugh A and volume of remnant right lobe is sufficient. Vascular exclusion technique is mandate for thrombectomy, whether total hepatic vascular exclusion, active *veno-venous* bypass, CPB are decided upon the condition and location of TT and classification by Li et al^[17]^ serve as a guide for surgical treatment. We reported HCC with Type III atrial thrombus along with 3 HVs and portal veins involvement, therefore, cardiopulmonary bypass was carried out despite of adverse effect of CPB such as coagulation abnormality, postoperative complication. However, the patient recovered well after surgery and no CPB related complications were experienced.

The reported morbidity and mortality were higher for *ante-situm* resection than conventional hepatectomy,^[18]^ however, recent advances in hepatic has greatly improved postoperative outcomes with appropriate hepatic vascular control technique and good hepatic reserve.^[10]^ Several large-scale studies have reported promising long-term survival of hepatectomy with thrombectomy in such an advanced group of patients. Reported mortality who underwent curative radical resection varied 0–9.9%, and median overall survival times varied from 16.4–30.8 months.^[8]^ Of note, the median survival times is about 2–5 months without treatment,^[19]^ and 10.7 months of patients with sorafenib alone which is lower than radical surgery.^[20]^ The postoperative recurrence and metastasis such as intrahepatic and lung metastasis are the most influencing factor that associates with poor prognosis in such patients even after radical surgery. Besides, the potential role of surgery combined targeted and immunotherapy might be a helpful for improving the long-term outcomes in advanced cases.

*Ante-situm* resection and liver transplantation as an extreme radical resection technique could be applied in the clinical practice of HCC patients with thrombi in IVC, right atrium, and portal vein. The long-term outcomes should be critically considered.

## Author contributions

**Conceptualization:** Tuerhongjiang Tuxun.

**Data curation:** Shadike Apaer, Gang Yao, Zhi-peng Wang, Shen-sen Gu, Qi Zeng, Aidan Aizezijiang, Jing Wu, Nuerzhatijiang Anweier.

**Supervision:** Jin-ming Zhao.

**Writing – original draft:** Tuerhongjiang Tuxun.

**Writing – review & editing:** Tuerhongjiang Tuxun, Jin-ming Zhao, Tao Li.
